# Repair of non-traumatic femoral head necrosis by marrow core decompression with bone grafting and porous tantalum rod implantation

**DOI:** 10.12669/pjms.36.6.2176

**Published:** 2020

**Authors:** Keyun Peng, Yu Wang, Jifeng Zhu, Chengling Li, Ziming Wang

**Affiliations:** 1 Keyun Peng, Orthopedic Central Joint and Limb Surgery, Army Medical Center of PLA (Daping Hospital), Army Medical University, Yuzhong District, Chongqing, China; 2 Yu Wang, Orthopedic Central Joint and Limb Surgery, Army Medical Center of PLA (Daping Hospital), Army Medical University, Yuzhong District, Chongqing, China; 3 Jifeng Zhu, Orthopedic Central Joint and Limb Surgery, Army Medical Center of PLA (Daping Hospital), Army Medical University, Yuzhong District, Chongqing, China; 4 Chengling Li, Orthopedic Central Joint and Limb Surgery, Army Medical Center of PLA (Daping Hospital), Army Medical University, Yuzhong District, Chongqing, China; 5 Ziming Wang, Orthopedic Central Joint and Limb Surgery, Army Medical Center of PLA (Daping Hospital), Army Medical University, Yuzhong District, Chongqing, China

**Keywords:** Bone grafting, Femoral head necrosis, Harris score, Marrow core decompression, Tantalum metal

## Abstract

**Objective:**

To compare the clinical effects of marrow core decompression with bone grafting and marrow core decompression with porous tantalum rod implantation in treating avascular necrosis of non-traumatic femoral head.

**Methods:**

This prospective study selected 60 patients (74 hips) with avascular necrosis of femoral head admitted to Daping Hospital from January 2018 to March 2019. According to treatment methods, the 60 patients were randomly divided into two groups, i.e. 30 patients in one group were treated by marrow core decompression with bone grafting, and the other 30 patients in the other group were treated with marrow core decompression and porous tantalum rod implantation.

**Results:**

All implantation treatments were successful. No significant difference was found in surgical duration, hemorrhage volume and duration of hospitalization stay between the two groups during follow-up. All Harris scores were significantly improved (P<0.05) following treatment compared to those before treatment. The Harris score of patients treated with porous tantalum rod implantation was higher than that of patients treated with bone grafting (P<0.05) after 12 months following treatment and such a difference was significant.

**Conclusion:**

The combination of marrow core decompression and porous tantalum rod implantation can better improve the functions of hip joints with early femoral head necrosis than marrow core decompression with bone grafting, and can also prevent articular cartilage from collapsing gradually.

## INTRODUCTION

Femoral head necrosis is a common orthopedic disease with an extremely high disability rate, where there is interruption of the blood supply or damage to the femoral head resulting in death of bone cells and bone marrow components with subsequent repair, further leading to femoral head structural changes, femoral head collapse and joint dysfunction, finally leading to arthritis.^1^ Osteonecrosis of the femoral head is also referred to as avascular necrosis and a common refractory disorder in the orthopedics field.^2^ Femoral head osteonecrosis can be divided into two major categories, traumatic and non-traumatic; the former is largely caused by hip traumas such as fracture of neck of femur and dislocation of hip joint, and the latter by excessive use of corticosteroids and intemperance.^3^ An early hip preservation surgery has been the main approach for the current treatment, including marrow core decompression, transplantation of fibula with blood vessels, transplantation of quadratus femoris muscle bone flap with pedicle.^4^ The technique of porous tantalum rod implantation has been applied in clinical practice and achieved good curative effects in recent years.^5^ We herein aimed to compare the clinical effects of marrow core decompression with bone grafting and marrow core decompression with porous tantalum rod implantation in patients with ischemic necrosis of non-traumatic femoral head.

## METHODS

Sixty patients with avascular necrosis of femoral head (74 hips) admitted to our hospital from January 2018 to March 2019 were selected, including 32 males and 28 females aged 25-65 years old, averaged at (46.7±13.9). Based on staging of pathological changes by the Association Research Circulation Osseous (ARCO), all patients belong to ARCO Stage I and II. According to treatment methods, the 60 patients were randomly divided into two groups, i.e. 30 patients (38 hips) treated with marrow core decompression with bone grafting were set as a bone grafting group and the other 30 patients (38 hips) treated with marrow core decompression and porous tantalum rod (Zimmer, USA) implantation were set as a porous tantalum rod group.

This study was approved by the ethics committee of our hospital at January 4^th^, 2018 (Approval No. AMCPLA201801003), and written informed consents have been obtained from all cases.

### Diagnostic criteria

1. With a long history of alcohol drinking or steroid hormone administration.
2. Pain felt in hip joints and inguinal regions and aggravated when standing or walking.
3. Positive Patrick sign and Thomas sign.
4. A definite diagnosis to be made based on hip joint X-ray and MRI examination.^6^

### Inclusion criteria

1. Those who had not received surgery for the affected hips.
2. Those who were aged 20-65.
3. Those who had early osteonecrosis of the femoral head (ARCO Stage I and II).

### Exclusion criteria

1. hose who had severe internal medical complications and cannot withstand surgery.
2. Those who had a body mass index of >40 kg/m^2^.
3. Those who suffered from pulmonary, urinary system and other infections.

### Preoperative preparation

All patients underwent routine examinations after admission to the hospital, including pelvis anteroposterior radiograph with lateral projection and MRI of the hip at the affected side. The osteonecrosis degree and staging were evaluated. Internal medical diseases were controlled actively to keep the levels of blood pressure and blood glucose, within a certain range, and surgery was performed as early as possible for elderly patients with such internal medical diseases.

### Internal fixation method

Marrow core decompression with bone grafting: Epidural anesthesia was used with the hip blocked up. A longitudinal incision was made on the external side of the femur 2 cm below the greater femur trochanter. Being monitored by the C-arm X-ray machine, the position of the guide pin was determined, and the guide pin was drilled into the center of the necrotic area beneath the femur head cartilage through femoral neck from under the trochanter and screwed into the edge of lesion area below the head using a decompressor with tube core. The biopsy device was screwed into the lesion area, and the yellowish white wax-like loose diseased tissues were taken out from the front end of the biopsy device and delivered for pathological examination. Then the outer sleeve of the decompressor was screwed out and the necrotic tissues within the femur head were completely removed with a spatula. The autologous bone graft from ilium was implanted into the focus decompression scraped area through the bone tunnel after being trimmed. Finally, an iliac strut graft was used to block the tunnel and secured by compression, and the incision was sutured.^7^

Marrow core decompression tantalum rod implantation: After the same anesthesia, incision and guide pin drilling processes as mentioned above, two hollow drill bits with the diameters of 8 mm and 9 mm were used successively to open the bone cortices on the external side of the femur along the guide pin. The drill bit was withdrawn when it reached approximately five mm of the subchondral bone through the femur neck. A special biopsy device with a diameter of six mm or seven mm was used for biopsy in the necrotic areas along the pinning passage. Finally, a 10 mm hollow drill was used to ream the marrow. Implantation materials of different lengths (80-120 mm which increased by five mm progressively) were used based on the measurements. The tunnel was tapped with a special screw tap, and the tantalum rod was screwed until approximately five mm below the subchondral bone. The incision was irrigated and sutured layer by layer.^8^

### Treatment after internal fixation

Antibiotics were applied routinely to prevent postoperative infection. The patients were allowed to do functional exercise of hip and knee joints on the bed at the 3rd day after surgery, ambulate after one week, bear weight after 3-6 months gradually as appropriate and bear full weight after six months while prohibited to bear any weight within 3 months.

### Main observational indices

The surgical duration, hemorrhage volume, hospitalization stay length and pain index scores of the two groups were recorded. The treatment outcomes were assessed based on the Harris hip scores in postoperative three, six and 12 months. The total score is 100 points, with 44 points for pain, 47 points for function, 4 points for deformity and 5 points for joint motion. Pain was scored with the visual observation analogy method, ranging from 0 point to 10 points, and 0 point indicates no pain and 10 points indicate severe pain.

### Statistical analysis

The SPSS 17.0 statistical software package was used for analysis. The data was expressed with mean ± standard deviation (X¯± s). The paired t-test was used for comparison among groups. P<0.05 indicated that the difference was significant.

## RESULTS

The two groups had comparable baseline clinical data such as age, gender and course of disease (P>0.05) (Table-I). The follow-up period was 12 months after surgery. Results analysis was conducted for 60 patients in total, among which 30 patients from the bone grafting group and 30 patients from the tantalum rod group. Harris scores between the two groups of patients before replacement had no significant difference (P>0.05). The Harris scores of the two groups after replacement were significantly improved compared with those before surgery. Harris scores of the two groups had significant difference 12 months after replacement (P<0.05) (Table-II).

**Table-I:** Baseline clinical data.

Item		Bone grafting group	Porous tantalum rod group
Gender	Male	17	15
	Female	13	15
Age	<45	13	12
	≥45	17	18
Position	Unilateral	24	22
	Bilateral	6	8
Trigger	Induced by steroid	16	17
	Intemperance	8	6
	Miscellaneous	6	7
ARCO Stage	Stage I	11	13
	Stage II	19	17

**Table-II:** Harris scores before and after treatment.

Group	Before surgery	3 months after surgery	6 months after surgery	12 months after surgery
Bone grafting	66.1±3.2	82.3±3.6#	95.1±3.7#	83.6±3.5#
Porous tantalum rod	66.8±3.1	83.1±3.4#	96.4±3.8#	95.8±3.7*#

* P<0.05 compared with the bone grafting group and #P<0.05 compared with those before surgery.

**Fig.1:**
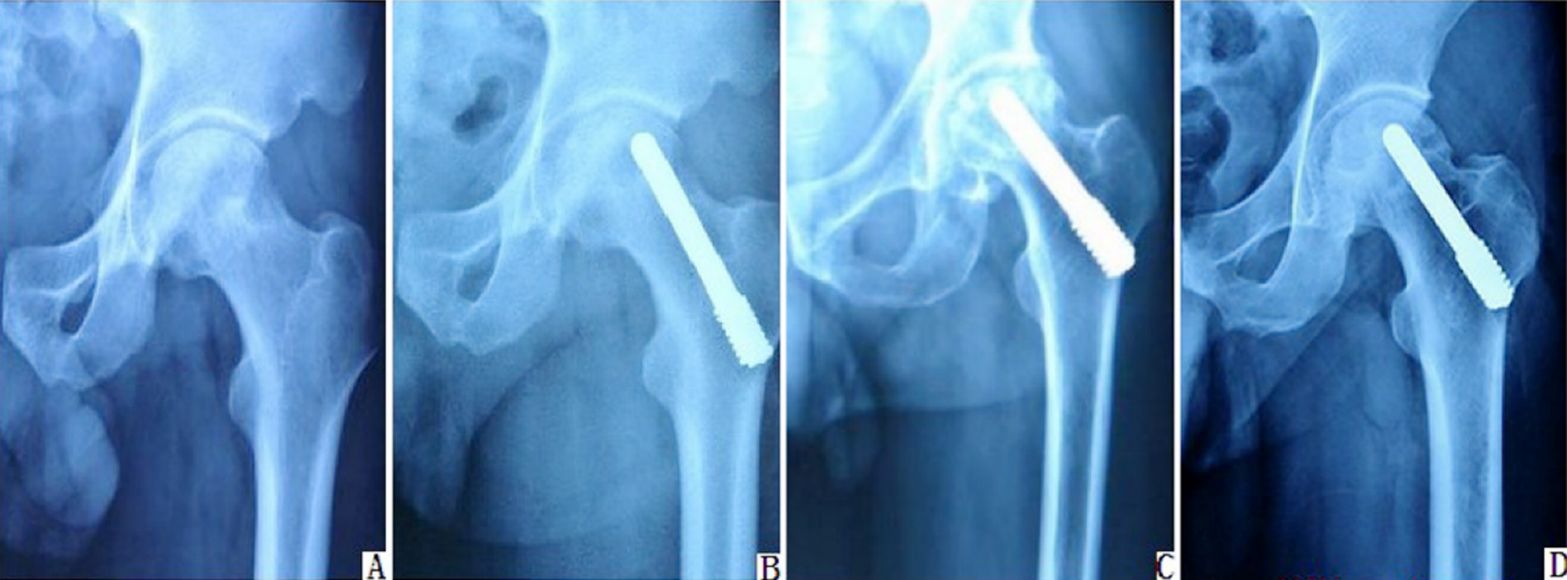
X-ray appearances for a male patient with Stage-II femoral head necrosis before and after treatment. A: Weight bearing area of femoral head exhibited cystic degeneration before surgery; B: X-ray appearance of surgical site 3 months after surgery; C: X-ray appearance of surgical site 6 months after surgery; D: X-ray appearance of surgical site 12 months after surgery.

Pain index scores between the two groups of patients before replacement had no significant difference (P>0.05). The pain index scores of the two groups after replacement had no difference 3 and 6 months after replacement (P>0.05). The pain index scores of the two groups had significant difference 12 months after replacement (P<0.05) (Table-III). The two groups of patients had no significant difference among surgical duration, hemorrhage volume or hospitalization stay length (P>0.05) (Table-IV). A 52-year-old male patient with Stage-II femoral head necrosis underwent treatment of porous tantalum rod implantation. The X-ray presented that the patient’s femoral head collapse was significantly mitigated 12 months after treatment (Fig.1).

**Table-III:** Pain index scores before and after surgery.

Group	Before surgery	3 months after surgery	6 months after surgery	12 months after surgery
Bone grafting	6.23±0.85	5.87±0.62	4.27±0.55	4.94±0.58
Porous tantalum rod	6.18±0.88	5.73±0.61	4.98±0.51	3.17±0.42*

* P<0.05 compared with the bone grafting group.

**Table-IV:** Surgical duration, hemorrhage volume and hospitalization stay length.

Group	Surgical duration (min)	Hemorrhage volume (ml)	Hospitalization stay length (d)
Bone grafting	73.2±12.8	124.7±20.3	12.1±2.4
Porous tantalum rod	70.6±12.5	119.8±18.4	10.9±2.2

## DISCUSSION

Currently, the commonly used surgical methods for femoral head necrosis suffer from the disadvantages of large operative wounds, high technical requirements and excessive complications after implantation.^9^ Although marrow core decompression can decrease intraosseous pressure, alleviate bone marrow edema and improve blood supply for femoral head, it cannot repair the femoral head, which lowers its biological strength and causes collapse.^10^ Guo et al. reported that femoral head collapse should be treated focusing on local effective mechanical support by modifying bone remodeling in the femoral head.^11^

It has been more than half a century since porous tantalum was applied in medical science. Tantalum rods have superior strength, fatigue properties, biocompatibility and initial stability for bones to those of natural osseous grafts, and they have low cytotoxicity and bacterial adhesion force.^12^ Such characteristics determine that the implantation of porous tantalum can not only provide safe and effective mechanical support for femoral head and subchondral bone lamellas, but also strengthen revascularization of necrotic areas and lower stress shielding, thus ensuring growth of bone into the necrotic zones.^13^ A meta-analysis showed that core decompression in combination with tantalum rod implantation gave satisfactory clinical results.^14^ Auregan et al. found that the mechanical support for femoral head may be improved through core decompression and insertion of a tantalum rod.^15^ Moreover, Moya-Angeler et al. reported that porous tantalum implants combined with core decompression provided structural support, without causing autograft harvest or infectious complications of bone allograft. However, the functional and clinical outcomes of this technique should be evaluated by long-term follow-up.^16^

No significant difference was present in surgical duration, hemorrhage volume, hospitalization stay length between the two groups of marrow core decompression with bone grafting and porous tantalum rod implantation in the study (P>0.05), indicating that both surgical methods were typified by simple operation, slight wound and less time consumption contributing to recovery of patients as early as possible. No significant difference existed between the preoperative Harris scores and pain index scores of the two groups (P>0.05) and the postoperative Harris scores of the two groups improve significantly compared with those before surgery (P<0.05). The Harris score of the porous tantalum rod group was significantly higher but the pain index score was lower than those of the bone grafting group 12 months after surgery (P<0.05), indicating that the porous tantalum rod implantation and the marrow core decompression with bone grafting had the same curative effects in early postoperative stage, can alleviate the degree of femoral head necrosis, feature simple operation and less time consumption.

Nevertheless, as time elapsed, in the group of marrow core decompression with bone grafting, bone resorption may be present and lead to femoral head collapse and the porous tantalum rod implantation can still provide strong support, promote revascularization of the necrotic areas, and delay gradual collapse of articular cartilage.^17^ The treatment outcomes are comparable to those in previous literatures.^14^,^18^,^19^ The satisfactory results herein can be ascribed to strict selection of surgical indications, proper location of tantalum rod, timely elimination of necrotic tissues in the anterior upper load-bearing area of the femoral head, gentle surgical operations and appropriate postoperative protection.

### Limitations of the study

First, the sample size is small. Second, the follow-up period may not be long enough. Further in-depth studies with larger sample size and longer follow-up are going in our group.

## CONCLUSION

The clinical effects of marrow core decompression with bone grafting and porous tantalum rod implantation on the ischemic necrosis of non-traumatic femoral head have seldom been compared hitherto. We herein found that the combination of marrow core decompression and porous tantalum rod implantation can better improve the functions of hip joints with early femoral head necrosis than marrow core decompression with bone grafting, and can also prevent articular cartilage from collapsing gradually.

### Authors’ Contributions:

**KP & YW:** Manuscript drafting, clinical data analysis.

**JZ & CL:** Clinical data collection and analysis.

**ZW:** Study design and significant manuscript revision.

**KP, YW, JZ, CL & ZW:** Approval of manuscript submission, responsible and accountable for the accuracy or integrity of this work.
